# A simplified frailty assessment using three objective measures predicts mid-term outcomes after cardiac surgery

**DOI:** 10.1007/s11748-025-02233-z

**Published:** 2025-12-19

**Authors:** Tasuku Honda, Masato Ogawa, Hiroshi Inuki, Norimasa Kubo, Tokunari Aritoshi, Masayuki Shiba, Kazuto Ishimoto, Naoya Kida, Chika Sugimoto, Naomi Yagi

**Affiliations:** 1 Department of Cardiovascular Surgery, Hyogo Prefectural Harima-Himeji General Medical Center, 3-264, Kamiya-cho, Himeji, 670-8560 Japan; 2 Division of Rehabilitation Medicine, Hyogo Prefectural Harima-Himeji General Medical Center, Himeji, Japan; 3 Division of Rehabilitation Medicine, Kobe University Graduate School of Medicine, Kobe, Japan; 4 Division of Rehabilitation Medicine, Hyogo Prefectural Kakogawa Medical Center, Kakogawa, Japan; 5 Advanced Medical Engineering Research Institute, University of Hyogo, Himeji, Japan

**Keywords:** Frailty, Cardiac surgery, Prognosis, Risk stratification

## Abstract

**Objective:**

Frailty is a major risk factor for adverse outcomes following cardiac surgery, yet its routine clinical integration is hindered by the lack of a standardized, convenient assessment method. This study aimed to develop and validate a simplified frailty model using three objective measures: gait speed, serum albumin, and grip strength.

**Methods:**

In this prospective observational study of 261 patients (≥ 65 years) undergoing elective cardiac surgery, frailty was assessed using both the Japanese Cardiovascular Health Study criteria and our simplified model. The model defined frailty as having ≥ 2 of the following: slowness (gait speed < 1.0 m/s), hypoalbuminemia (albumin ≤ 3.5 g/dL), and weakness (grip strength < 28 kg for men, < 18 kg for women).

**Results:**

The simplified model demonstrated high diagnostic accuracy for frailty defined by the Japanese Cardiovascular Health Study criteria (area under the curve = 0.868; sensitivity, 55.8%; specificity, 91.4%). Frailty defined by our model was a strong predictor of worse 3-year survival (hazard ratio, 10.43; 95% confidence interval, 2.82–38.58; *p* < 0.001) and event-free survival (hazard ratio, 2.52; 95% confidence interval, 1.47–4.34; *p* < 0.001), with prognostic power comparable to the Japanese Cardiovascular Health Study criteria.

**Conclusions:**

A simplified frailty model incorporating gait speed, serum albumin, and grip strength provides robust diagnostic and prognostic utility. Its objectivity and ease of use may facilitate consistent preoperative risk stratification in patients undergoing cardiac surgery.

**Supplementary Information:**

The online version contains supplementary material available at 10.1007/s11748-025-02233-z.

## Introduction

Frailty is a significant prognostic factor in the preoperative evaluation for cardiac surgery [1] and is strongly correlated with the risk of major adverse cardiac and cerebrovascular events (MACCE) [2]. We also reported that pre-frailty, as well as frailty, has an adverse effect on the mid-term prognosis after cardiac surgery [3].

Despite its recognized importance, the lack of a standardized, easily applicable frailty assessment tool has limited its consistent establishment as a predictor. One reason for this is the wide variety of procedures for assessing frailty. Multiple frailty measurements exist, but their quality varies, and there is no international standard for measuring frailty [4]. Another reason is the complexity of the assessment process itself. In 2024, the European Society of Cardiology (ESC) published a statement on pre-interventional frailty assessment in patients scheduled for cardiac surgery or transcatheter aortic valve implantation (TAVI) [5].

The ESC statement emphasized slowness (5-m gait speed < 0.8 m/s) and hypoalbuminemia (albumin ≤ 3.5 g/dL) as the initial assessment of frailty because numerous studies have reported that these two parameters can highly predict important clinical outcomes such as mortality and rehospitalization in patients undergoing cardiac surgery or TAVI. Both are elements included in many comprehensive frailty assessment tools and essential components of the assessment in the Consensus Statement figure.

While this guidance is invaluable for initial risk stratification, it often necessitates further assessment using more comprehensive but less convenient traditional instruments like the Fried criteria [6] or essential frailty tool (EFT) [7] to confirm frailty status. This multi-step process, while thorough, can be inconvenient in busy clinical settings and highlights an unmet need for a more streamlined, yet sufficiently comprehensive, frailty assessment that can be readily implemented.

To construct such a tool based on the ESC's core recommendations, we incorporated a third objective measure. Weakness, assessed by grip strength, is one of the most powerful indicators of frailty assessment, as are slowness and hypoalbuminemia. It is a core component of many established frailty indices, including the Cardiovascular Health Study (CHS) criteria [6], and offers an objective, quantitative assessment less prone to subjective patient interpretation or observer variability.

This study primarily aimed to design a simple, three-item frailty assessment model, incorporating slowness, hypoalbuminemia, and weakness, that is easy to use in clinical practice and to statistically evaluate its diagnostic performance in identifying frailty as defined by the J-CHS criteria. As a secondary aim, we sought to compare the impact of frailty, diagnosed by this simplified model, on the mid-term prognosis after cardiac surgery with frailty diagnosed using the standard J-CHS assessment within the same study cohort.

## Methods

### Study design and subjects

This analysis utilized a patient cohort from a prospective, single-center observational study conducted at the Hyogo Brain and Heart Center, Japan. The study, which adhered to the STROBE guidelines and the Declaration of Helsinki, was approved by the institutional Research Ethics Committee (protocol R3-27). In brief, between August 2018 and November 2021, we enrolled patients aged 65 years or older scheduled for elective open-heart surgery via median sternotomy. Patients were excluded if they underwent emergency surgery, specific aortic procedures requiring a left thoracotomy, had pre-existing functional walking difficulties, or had missing data. All participants provided informed consent, with an opt-out option available. Postoperative follow-up, conducted via medical records and telephone interviews, continued until January 2023.

### Frailty assessment

The reference standard for frailty was the Japanese version of the Cardiovascular Health Study (J-CHS) index [8]. According to this index, frailty is defined by the presence of at least three of the five following components: significant weight loss (≥ 2 kg in 6 months), self-reported exhaustion, low physical activity, slowness (gait speed < 1.0 m/s), and weakness (grip strength < 28 kg for men or < 18 kg for women). Gait speed was measured over a 5-m course at the patient's usual pace. In line with this definition, individuals who met two or fewer criteria were categorized as non-frail.

In the present study, we developed a new three-item frailty assessment model consisting of slowness and hypoalbuminemia, which were the initial tests recommended in the ESC statements [4], plus weakness. To align with the syndromic nature of frailty and to optimize diagnostic performance against the J-CHS criteria, patients meeting two or more of these three criteria were classified as “frailty,” while the others were classified as “non-frailty.” Supplement Fig. 1 shows comparison of standard and simplified frailty assessment. Incidentally, sarcopenia, which represents physical frailty, was diagnosed based on the criteria reported by the Asian Working Group [9].

### Mid-term prognosis after cardiac surgery

We compared survival and event-free rates for 3 years after cardiac surgery in two groups, dividing them according to the presence or absence of frailty diagnosed by our simple assessment model. The above results were compared with the standard assessment tool (J-CHS) to classify frailty.

### Statistical analysis

This study was exploratory in nature, and formal sample size calculations were not performed prior to study initiation; however, the sample size was determined by the number of eligible patients undergoing surgery during the study period.

Baseline characteristics were summarized using descriptive statistics. Continuous variables were presented as median [interquartile range] and categorical variables as number (%). To evaluate the performance of the simplified assessment model in diagnosing frailty as defined by the J-CHS criteria (the reference standard), we conducted the following statistical analyses:

1. Development and Selection of Simplified Frailty Models: We designed three predictive models for assessing frailty (Model 1: slowness alone, Model 2: slowness and hypoalbuminemia, and Model 3: slowness, hypoalbuminemia, and weakness). For each model, we calculated each model's diagnostic accuracy (sensitivity, specificity, positive predictive value [PPV], and negative predictive value [NPV]) using the J-CHS classification as the reference. We then calculated the area under the receiver operating characteristic (ROC) curve (AUC) from each candidate model. These AUCs were compared using the DeLong test, and the model demonstrating the most suitable diagnostic performance for identifying J-CHS-defined frailty was selected for further analysis.
2. Assessment of Component Contribution within the Selected Model: For the selected model (model 3), we further assessed the contribution of its individual components (slowness, hypoalbuminemia, weakness) to the identification of J-CHS-defined frailty characteristics. This involved examining the incremental changes in AUC, as well as calculating the net reclassification improvement (NRI) and integrated discrimination improvement (IDI) to quantify the improvement in classification accuracy for J-CHS-defined frailty status when adding components. It is important to note that this NRI/IDI analysis was specifically focused on evaluating the incremental improvement in diagnosing J-CHS-defined frailty by adding components to the simplified model, and not comparing the overall prognostic utility of the simplified model versus the J-CHS criteria for predicting clinical outcomes using these specific metrics in this step.

To evaluate the prognostic utility of the frailty models, we compared cumulative event rates for all-cause mortality and morbidity (defined as all-cause mortality and MACCE-related rehospitalization) using the log-rank test. These differences were visualized with Kaplan–Meier curves. We used multivariable Cox proportional hazards models to calculate hazard ratios (HRs) adjusted for clinically relevant covariates, including age, gender, and body mass index. The proportional hazards assumption for the Cox models was verified by examining log-minus-log survival plots and testing Schoenfeld residuals.

All statistical analyses were performed using the EZR version 1.68 [10]. All p-values were two-sided, and p values of < 0.05 were considered to indicate statistical significance. Given the exploratory nature of some analyses, p-values are presented without formal adjustment for multiple comparisons and should be interpreted accordingly.

## Results

The final analysis included 261 patients (median age, 73 years; 30% women) who completed a preoperative frailty assessment prior to elective cardiac surgery. The patient cohort for this study has been described in detail in our previous publication [3]. The most common procedures were valve surgery (n = 106) and aortic surgery (n = 106), followed by coronary artery bypass grafting (n = 43). Application of the standard J-CHS criteria resulted in 86 patients (33%) being classified as frail, while the remaining 175 (67%) were classified as non-frail. During the observation period, there were 13 mortalities. The etiologies included cerebrovascular disease in 3 patients, pneumonia in 2, and one patient each for heart failure, aortic dissection, cancer, and sepsis; the cause of death remained indeterminate for four patients.

### Development and validation of simplified frailty assessment models to diagnose J-CHS-defined frailty

We evaluated the validity of the simplified assessment models as predictive models for identifying frailty as defined by the J-CHS criteria. The diagnostic accuracy of these models is presented in Table 1. Diagnostic accuracy improved with the addition of variables from Model 1 (slowness alone) to Model 3 (slowness, hypoalbuminemia, and weakness). Model 3 achieved the highest diagnostic accuracy (79.7%), with a sensitivity of 55.8% and a specificity of 91.4%. While Model 2 (slowness and hypoalbuminemia) showed high sensitivity (94.3%), its specificity was considerably lower (18.6%), resulting in a lower overall diagnostic accuracy (69.3%) compared to Model 1 (71.6%) and Model 3. Model 3 also demonstrated the highest positive likelihood ratio (6.512). Supplement Fig. 2 shows the ROC curves for each simplified assessment model. Consistent with the diagnostic accuracy findings, the AUC increased with each additional variable: Model 1 (AUC: 0.732, 95% CI: 0.676–0.788), Model 2 (AUC: 0.769, 95% CI: 0.706–0.823), and Model 3 (AUC: 0.868, 95% CI: 0.825–0.911) (Table 2). The AUC for Model 3 was significantly higher than that for Model 1 and Model 2 (p < 0.001 for both comparisons).

**Table 1:** Diagnostic accuracy of each simplified frailty assessment model

	Model 1	Model 2	Model 3
sensitivity	68.6%	94.3%	55.8%
specificity	77.9%	18.6%	91.4%
positive predictive value	86.3%	70.2%	76.2%
negative predictive value	54.9%	61.5%	80.8%
diagnostic accuracy	71.6%	69.3%	79.7%
positive likelihood ratio	3.104	1.158	6.512
negative likelihood ratio	0.403	0.307	0.483

The variables for each model are as follows

Model 1: slowness Model 2: slowness, hypoalbuminemia Model 3: slowness, hypoalbuminemia, weakness

**Table 2:** Evaluation of the predictive ability of each simplified frailty assessment model

	AUC	95% CI	*p* value	NRI	95% CI	*p* value	IDI	95% CI	*p* value
Model 1	0.732	0.676–0.788							
Model 2	0.769	0.706–0.823	p < 0.001	0.353	0.139–0.567	0.001	0.027	0.002–0.051	0.036
Model 3	0.868	0.825–0.911	p < 0.001	0.794	0.704–0.884	p < 0.001	0.210	0.152–0.268	p < 0.001

The variables for each model are as follows

Model 1: slowness Model 2: slowness, hypoalbuminemia Model 3: slowness, hypoalbuminemia, weakness

AUC: Area Under the Curve, CI: Confidence Interval, NRI: Net Reclassification Improvement, IDI: Integrated Discrimination Improvement

The contribution of each component to Model 3 was further assessed (Table 2). The sequential addition of hypoalbuminemia to Model 1 (slowness) to create Model 2, and subsequently weakness to Model 2 to create Model 3, resulted in significant improvements in both Net Reclassification Improvement (NRI) and Integrated Discrimination Improvement (IDI) at each step (all p < 0.01). Specifically, adding hypoalbuminemia yielded an NRI of 0.353 (95% CI: 0.139–0.567) and an IDI of 0.027 (95% CI: 0.002–0.051). Further adding weakness to create Model 3 resulted in a substantial NRI of 0.794 (95% CI: 0.704–0.884) and an IDI of 0.210 (95% CI: 0.152–0.268) when compared to Model 1, highlighting the significant contribution of each of these three variables to the model's ability to identify J-CHS-defined frailty.

Based on these findings, Model 3 was selected as the simplified frailty assessment for further comparison. Using Model 3, 63 patients (24.1%) were classified as frail, compared to 86 patients (33.0%) classified as frail by the J-CHS criteria.

### Baseline characteristics according to frailty status by simplified model (Model 3) and standard J-CHS criteria

Supplement Table 1 through 3 shows the baseline characteristics and perioperative data of patients grouped by frailty status, as defined by the selected simplified assessment (Model 3) and the standard J-CHS criteria.

When assessed by either Model 3 or the J-CHS criteria, frail patients were significantly older and more likely to have lower body mass index, sarcopenia (although SMI p = 0.113 for Model 3), hypoalbuminemia, malnutrition, weight loss, exhaustion, low activity, slowness, and weakness compared to non-frail patients (all p < 0.05). The EuroSCORE II, a predictor of mortality after cardiac surgery, was significantly higher in frail patients defined by J-CHS criteria (p = 0.050) but did not reach statistical significance for Model 3-defined frailty (p = 0.083). Notably, when using the simplified assessment (Model 3), there were no significant differences in gender distribution or SMI between frail and non-frail patients (p = 0.756 and p = 0.113, respectively). In contrast, using the J-CHS criteria, frail patients included a significantly higher proportion of females (p < 0.001) and had significantly lower SMI (p < 0.001).

### Prognostic value of the selected simplified frailty assessment model (Model 3)

**Fig. 1:**
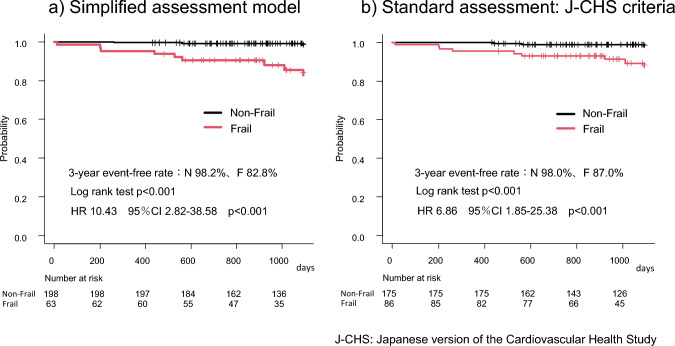
Kaplan–Meier curves for all-cause death by each frailty assessment. (**a**) Simplified assessment model (**b**) Standard assessment: J-CHS criteria

**Fig. 2:**
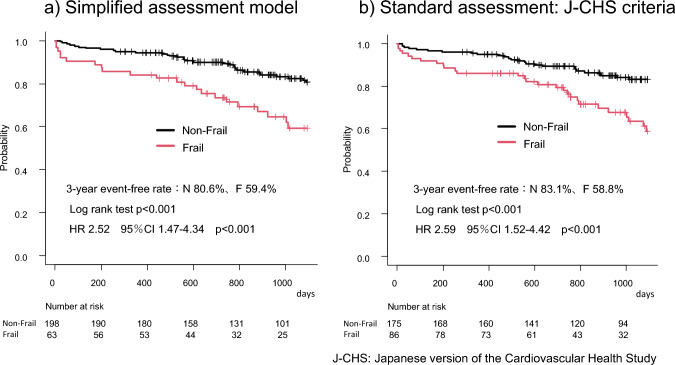
Kaplan–Meier curves for all-cause death and readmission related to major adverse cardiovascular and cerebrovascular events by each frailty assessment. (**a**) Simplified assessment model (**b**) Standard assessment: J-CHS criteria

We then compared the 3-year outcomes after cardiac surgery for patients classified by our simplified assessment (Model 3) and by the standard J-CHS criteria. Figure 1a shows the Kaplan–Meier curves for survival rates after 3 years of cardiac surgery for frailty and non-frailty patients classified by simplified assessment. The 3-year survival rates were 82.8, and 98.2% for the frailty and non-frailty groups, respectively (log-rank test: p < 0.001). Compared to the non-frailty group, there was an increase in adjusted mortality among patients in the frailty (HR, 10.43; 95% CI, 2.82–38.58, p < 0.001). This result was comparable to the 3-year survival rates observed when using the standard J-CHS criteria for classification (Fig. 1b). Next, Kaplan–Meier curves for the composite event of all-cause death and MACCE-related rehospitalization are shown in Fig. 2a and b for frailty and non-frailty patients classified by Model 3. The 3-year event-free rates were 59.4, and 80.6% for the frailty and non-frailty groups, respectively (log-rank test: p < 0.001). There was an increased adjusted morbidity in the frailty compared with the non-frailty group (HR, 2.59; 95% CI, 1.52–4.42, p < 0.001). As shown on Fig. 2b, this result was comparable to the event-free rate 3 years after surgery for both groups classified as with or without frailty by J-CHS criteria.

## Discussion

The present study demonstrated that a simple frailty assessment model consisting of three objective variables—gait speed, serum albumin level, and grip strength—showed reasonable diagnostic performance for identifying frailty defined by the J-CHS criteria and was significantly associated with mid-term mortality and morbidity after cardiac surgery. While this simplified model showed slightly lower sensitivity than the standard J-CHS criteria, it offered comparable specificity and prognostic value, coupled with the significant advantage of using easily quantifiable and readily available clinical measures.

Previous studies have reported that each of the three objective variables in the simplified model is an independent predictor of cardiac surgery outcomes.

First, as for gait speed, a previous study reported that slow gait speed was associated with significantly higher mortality and morbidity rates one year after surgery, where each decline in 0.1 m/s of gait speed conferred a twofold increased risk of mortality [11]. Second, a meta-analysis examining the association between preoperative serum albumin levels and outcomes after cardiac surgery reported that hypoalbuminemia in patients undergoing cardiac surgery was associated with a higher rate of all-cause mortality and increased risk of complications [12]. Third, another study on grip strength and mortality after cardiac surgery found that preoperative grip weakness predicted 1-year and 30-day mortality with odds ratios of 2.44 (95% CI 1.39–4.29) and 2.83 (1.38–5.81), respectively [13]. These findings support the validity of our simplified assessment model, suggesting it can identify frailty with a prognostic accuracy comparable to that of standard models. Therefore, our three-item model potentially offers a more streamlined approach, possibly serving as a more comprehensive initial assessment or bridging the gap between initial screening and more cumbersome traditional tools.

In this study, the number of items used to diagnose frailty is fewer than in the standard assessment tools such as the J-CHS criteria. There has been discussion about how much frailty assessment can be simplified without losing its essence [14]. In the field of cardiac surgery, several studies have been reported that aim to facilitate the assessment of frailty. The EFT [7] and the Study of Osteoporotic Fractures (SOF) index [15] are representative of such studies. Our model distinguishes itself by exclusively using three objective, performance-based or laboratory measures, potentially reducing inter-rater variability compared to some components of other assessment tools.

The strength of our study is that all variables are quantified indices that can be easily measured. Serum albumin levels are routine laboratory tests before cardiac surgery. In addition, non-medical staff can measure gait speed and grip strength if a stopwatch and hand dynamometer are available. Recently, applications have emerged that use smartphones to record walking style and analyze the videos to determine gait speed and balance [16]. The simplicity and accessibility of this diagnostic approach are major strengths of this study. We anticipate that its adoption could promote the standardization of frailty assessment, ultimately improving prognostic evaluation and treatment strategy selection in cardiac surgery.

One of the interesting findings in this study was that, unlike the J-CHS criteria, our simplified assessment did not show significant differences in gender distribution or SMI between frail and non-frail patients. The simplified assessment model excludes the diagnostic items of the J-CHS criteria: exhaustion, low activity, and weight loss. A previous study investigating gender differences in heart failure frailty phenotypes reported that women were more likely than men to experience exhaustion (odds ratio 5.46, 95% CI 1.12–26.50, P = 0.035) and low activity (odds ratio 2.34, 95% CI 1.09–4.99, P = 0.028) as significantly higher [17]. It was also suggested that the exclusion of weight loss may have diminished the relevance of SMI. Finally, our simplified model serves as a practical tool to flag high-risk patients, not to exclude them from surgery outright, but to ensure they receive a more comprehensive "Heart Team" discussion, consideration of less invasive procedures, and an opportunity for risk mitigation through prehabilitation.

### Limitations

We acknowledge several limitations. First, the findings are derived from a single-center cohort, which may restrict their external validity. Future multicenter studies are needed to confirm our results in a broader population. Second, our simplified assessment tool is confined to physical domains and does not capture the cognitive or psychosocial aspects of frailty. This focus on physical performance may lead to an underestimation of the true prevalence of frailty.

It is also important to discuss the difference in cutoff values for gait speed. Gait speed is used as the main parameter in frailty evaluation, and its cutoff value is defined as 0.8 m/s in Europe and the U.S. and 1.0 m/s in Japan, with differences in reference values seen in each region. A meta-analysis reported that after adjusting for age and gender, the 5-m walking time of Japanese was approximately 0.40 s faster than that of non-Asians [18]. It is speculated that this is due to the possibility that the lean body mass of the Japanese is denser than that of Westerners, and to lifestyle habits such as living on tatami mats, which strengthens lower limb muscle strength and balance ability. Therefore, differences in gait speed by region and race should be fully considered in assessing frailty.

## Conclusions

In conclusion, this study demonstrates that a simple model incorporating gait speed, serum albumin, and grip strength is a useful tool for frailty assessment. This approach not only showed a strong correlation with standard J-CHS criteria but also effectively predicted mid-term prognosis after cardiac surgery. Given that these three variables can be easily and objectively measured, this model offers a highly versatile and practical method for routine preoperative risk stratification.

## Supplementary Information

Below is the link to the electronic supplementary material.

Supplementary file1 (PPTX 311 kb)
