# Supplementary material for: A simplified frailty assessment using three objective measures predicts mid-term outcomes after cardiac surgery
Source: Gen Thorac Cardiovasc Surg. 2025 Dec 19;74(6):581–7. doi: 10.1007/s11748-025-02233-z (PMC13219124; doi:10.1007/s11748-025-02233-z)
Supplement: Supplementary file 1 — Supplementary file1 (PPTX 311 kb) [file 11748_2025_2233_MOESM1_ESM.pptx]

## Slide 1
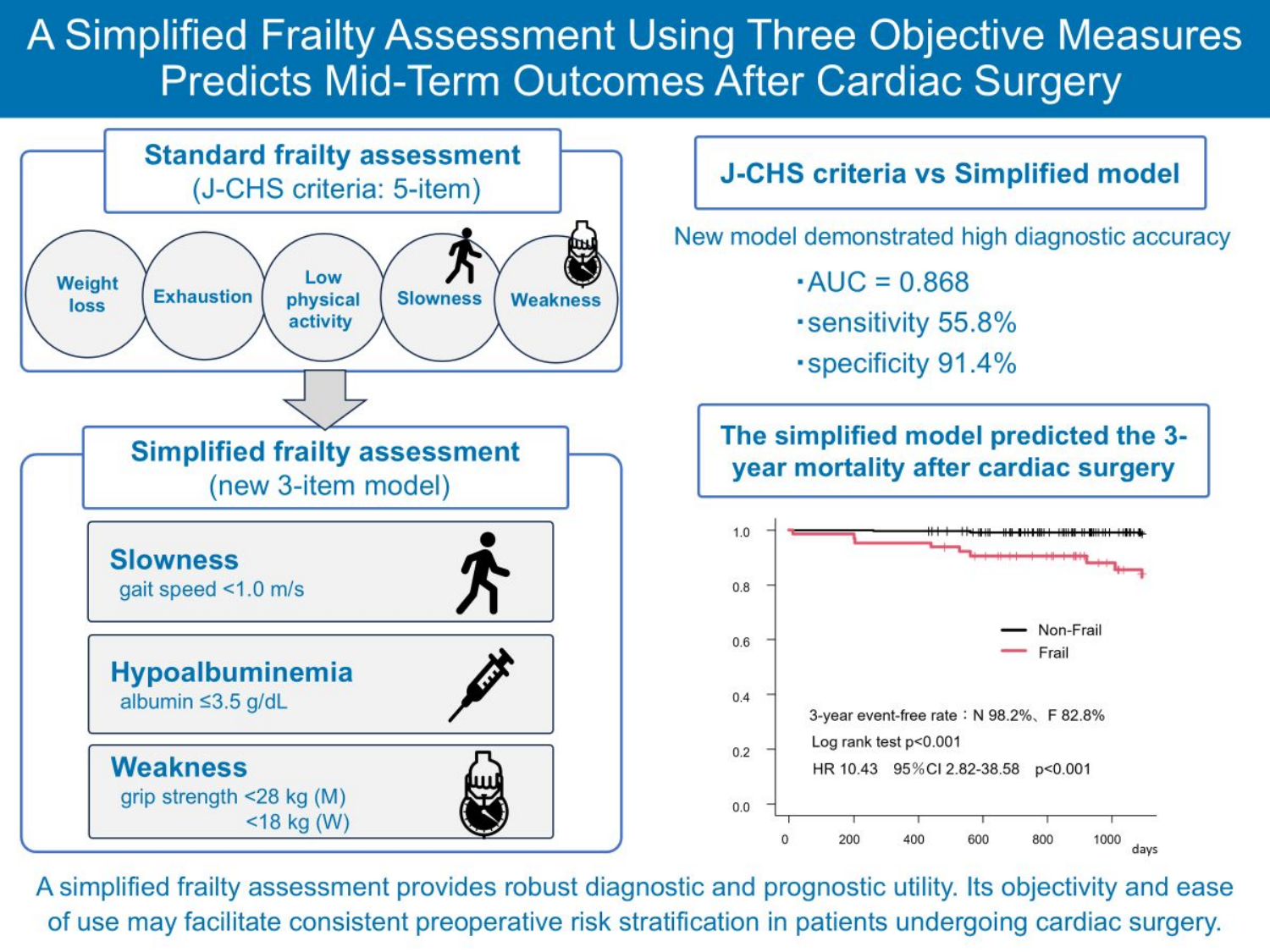

## Slide 2
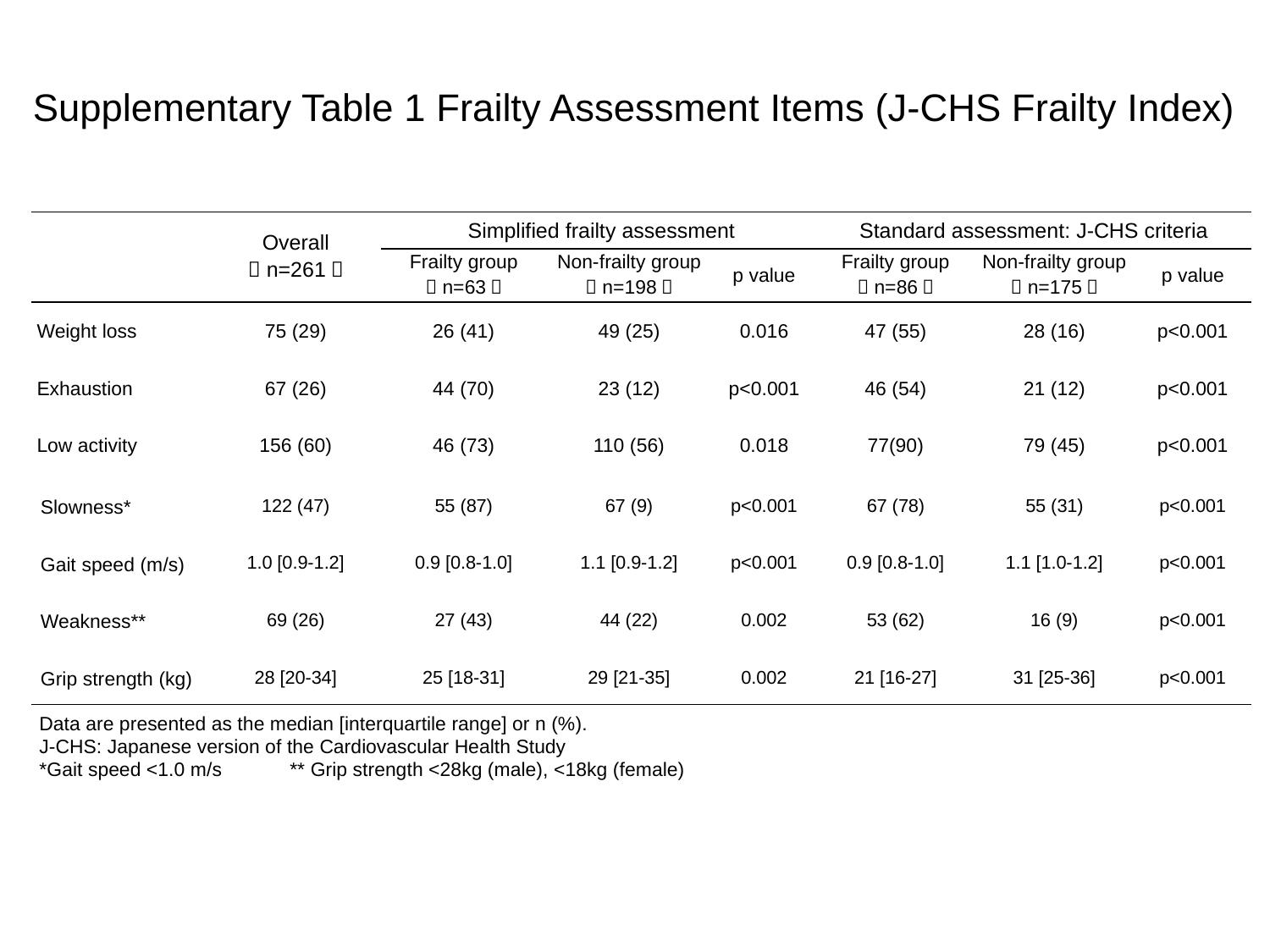

Supplementary Table 1 Frailty Assessment Items (J-CHS Frailty Index)
| | Overall （n=261） | Simplified frailty assessment | | | Standard assessment: J-CHS criteria | | |
| --- | --- | --- | --- | --- | --- | --- | --- |
| | | Frailty group （n=63） | Non-frailty group （n=198） | p value | Frailty group （n=86） | Non-frailty group （n=175） | p value |
| Weight loss | 75 (29) | 26 (41) | 49 (25) | 0.016 | 47 (55) | 28 (16) | p<0.001 |
| Exhaustion | 67 (26) | 44 (70) | 23 (12) | p<0.001 | 46 (54) | 21 (12) | p<0.001 |
| Low activity | 156 (60) | 46 (73) | 110 (56) | 0.018 | 77(90) | 79 (45) | p<0.001 |
| Slowness\* | 122 (47) | 55 (87) | 67 (9) | p<0.001 | 67 (78) | 55 (31) | p<0.001 |
| Gait speed (m/s) | 1.0 [0.9-1.2] | 0.9 [0.8-1.0] | 1.1 [0.9-1.2] | p<0.001 | 0.9 [0.8-1.0] | 1.1 [1.0-1.2] | p<0.001 |
| Weakness\*\* | 69 (26) | 27 (43) | 44 (22) | 0.002 | 53 (62) | 16 (9) | p<0.001 |
| Grip strength (kg) | 28 [20-34] | 25 [18-31] | 29 [21-35] | 0.002 | 21 [16-27] | 31 [25-36] | p<0.001 |
Data are presented as the median [interquartile range] or n (%).
J-CHS: Japanese version of the Cardiovascular Health Study
*Gait speed <1.0 m/s　　　** Grip strength <28kg (male), <18kg (female)

## Slide 3
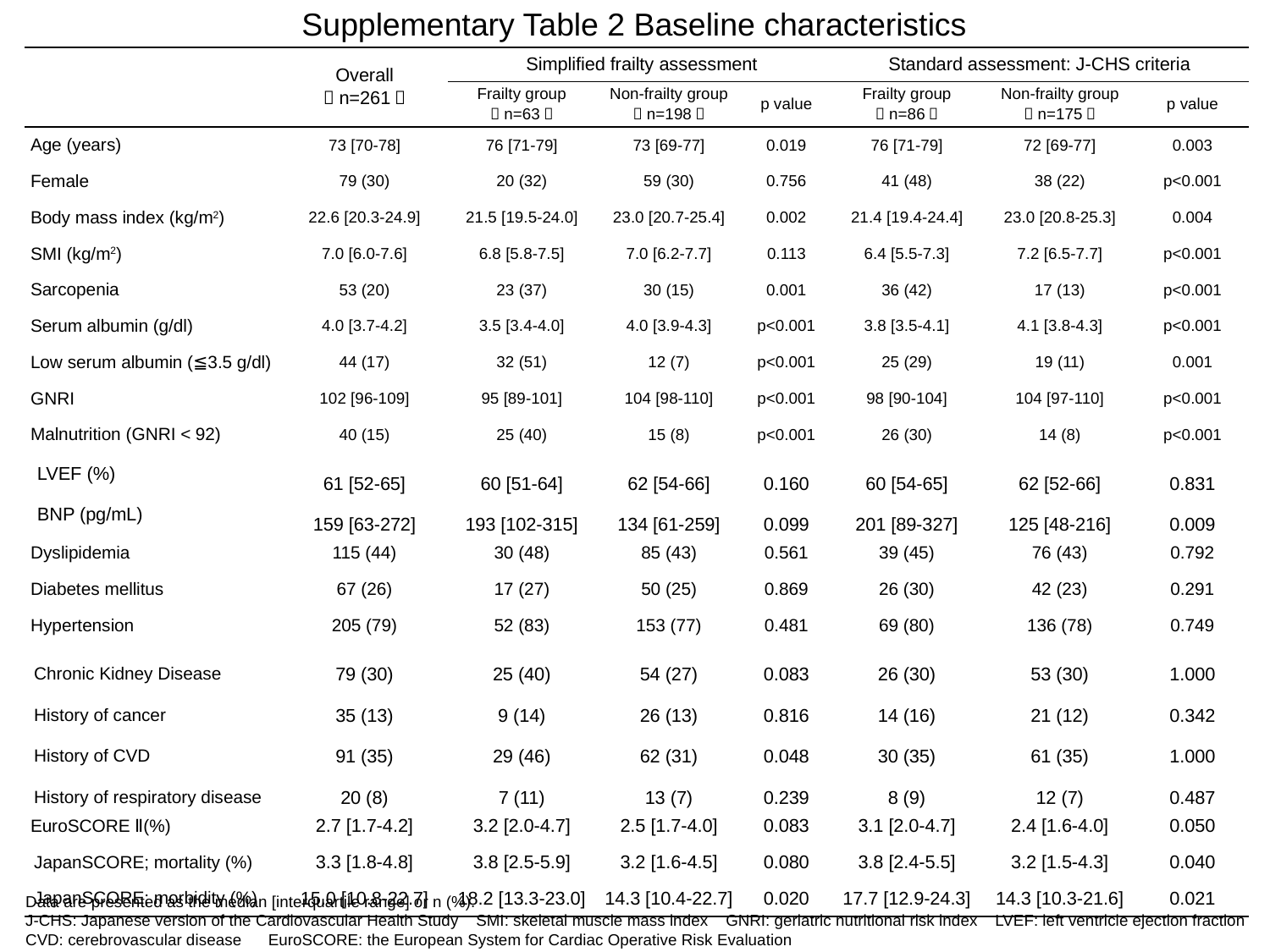

Supplementary Table 2 Baseline characteristics
| | Overall （n=261） | Simplified frailty assessment | | | Standard assessment: J-CHS criteria | | |
| --- | --- | --- | --- | --- | --- | --- | --- |
| | | Frailty group （n=63） | Non-frailty group （n=198） | p value | Frailty group （n=86） | Non-frailty group （n=175） | p value |
| Age (years) | 73 [70-78] | 76 [71-79] | 73 [69-77] | 0.019 | 76 [71-79] | 72 [69-77] | 0.003 |
| Female | 79 (30) | 20 (32) | 59 (30) | 0.756 | 41 (48) | 38 (22) | p<0.001 |
| Body mass index (kg/m2) | 22.6 [20.3-24.9] | 21.5 [19.5-24.0] | 23.0 [20.7-25.4] | 0.002 | 21.4 [19.4-24.4] | 23.0 [20.8-25.3] | 0.004 |
| SMI (kg/m2) | 7.0 [6.0-7.6] | 6.8 [5.8-7.5] | 7.0 [6.2-7.7] | 0.113 | 6.4 [5.5-7.3] | 7.2 [6.5-7.7] | p<0.001 |
| Sarcopenia | 53 (20) | 23 (37) | 30 (15) | 0.001 | 36 (42) | 17 (13) | p<0.001 |
| Serum albumin (g/dl) | 4.0 [3.7-4.2] | 3.5 [3.4-4.0] | 4.0 [3.9-4.3] | p<0.001 | 3.8 [3.5-4.1] | 4.1 [3.8-4.3] | p<0.001 |
| Low serum albumin (≦3.5 g/dl) | 44 (17) | 32 (51) | 12 (7) | p<0.001 | 25 (29) | 19 (11) | 0.001 |
| GNRI | 102 [96-109] | 95 [89-101] | 104 [98-110] | p<0.001 | 98 [90-104] | 104 [97-110] | p<0.001 |
| Malnutrition (GNRI < 92) | 40 (15) | 25 (40) | 15 (8) | p<0.001 | 26 (30) | 14 (8) | p<0.001 |
| LVEF (%) | 61 [52-65] | 60 [51-64] | 62 [54-66] | 0.160 | 60 [54-65] | 62 [52-66] | 0.831 |
| BNP (pg/mL) | 159 [63-272] | 193 [102-315] | 134 [61-259] | 0.099 | 201 [89-327] | 125 [48-216] | 0.009 |
| Dyslipidemia | 115 (44) | 30 (48) | 85 (43) | 0.561 | 39 (45) | 76 (43) | 0.792 |
| Diabetes mellitus | 67 (26) | 17 (27) | 50 (25) | 0.869 | 26 (30) | 42 (23) | 0.291 |
| Hypertension | 205 (79) | 52 (83) | 153 (77) | 0.481 | 69 (80) | 136 (78) | 0.749 |
| Chronic Kidney Disease | 79 (30) | 25 (40) | 54 (27) | 0.083 | 26 (30) | 53 (30) | 1.000 |
| History of cancer | 35 (13) | 9 (14) | 26 (13) | 0.816 | 14 (16) | 21 (12) | 0.342 |
| History of CVD | 91 (35) | 29 (46) | 62 (31) | 0.048 | 30 (35) | 61 (35) | 1.000 |
| History of respiratory disease | 20 (8) | 7 (11) | 13 (7) | 0.239 | 8 (9) | 12 (7) | 0.487 |
| EuroSCORE Ⅱ(%) | 2.7 [1.7-4.2] | 3.2 [2.0-4.7] | 2.5 [1.7-4.0] | 0.083 | 3.1 [2.0-4.7] | 2.4 [1.6-4.0] | 0.050 |
| JapanSCORE; mortality (%) | 3.3 [1.8-4.8] | 3.8 [2.5-5.9] | 3.2 [1.6-4.5] | 0.080 | 3.8 [2.4-5.5] | 3.2 [1.5-4.3] | 0.040 |
| JapanSCORE; morbidity (%) | 15.0 [10.8-22.7] | 18.2 [13.3-23.0] | 14.3 [10.4-22.7] | 0.020 | 17.7 [12.9-24.3] | 14.3 [10.3-21.6] | 0.021 |
Data are presented as the median [interquartile range] or n (%).
J-CHS: Japanese version of the Cardiovascular Health Study SMI: skeletal muscle mass index GNRI: geriatric nutritional risk index LVEF: left ventricle ejection fraction
CVD: cerebrovascular disease EuroSCORE: the European System for Cardiac Operative Risk Evaluation

## Slide 4
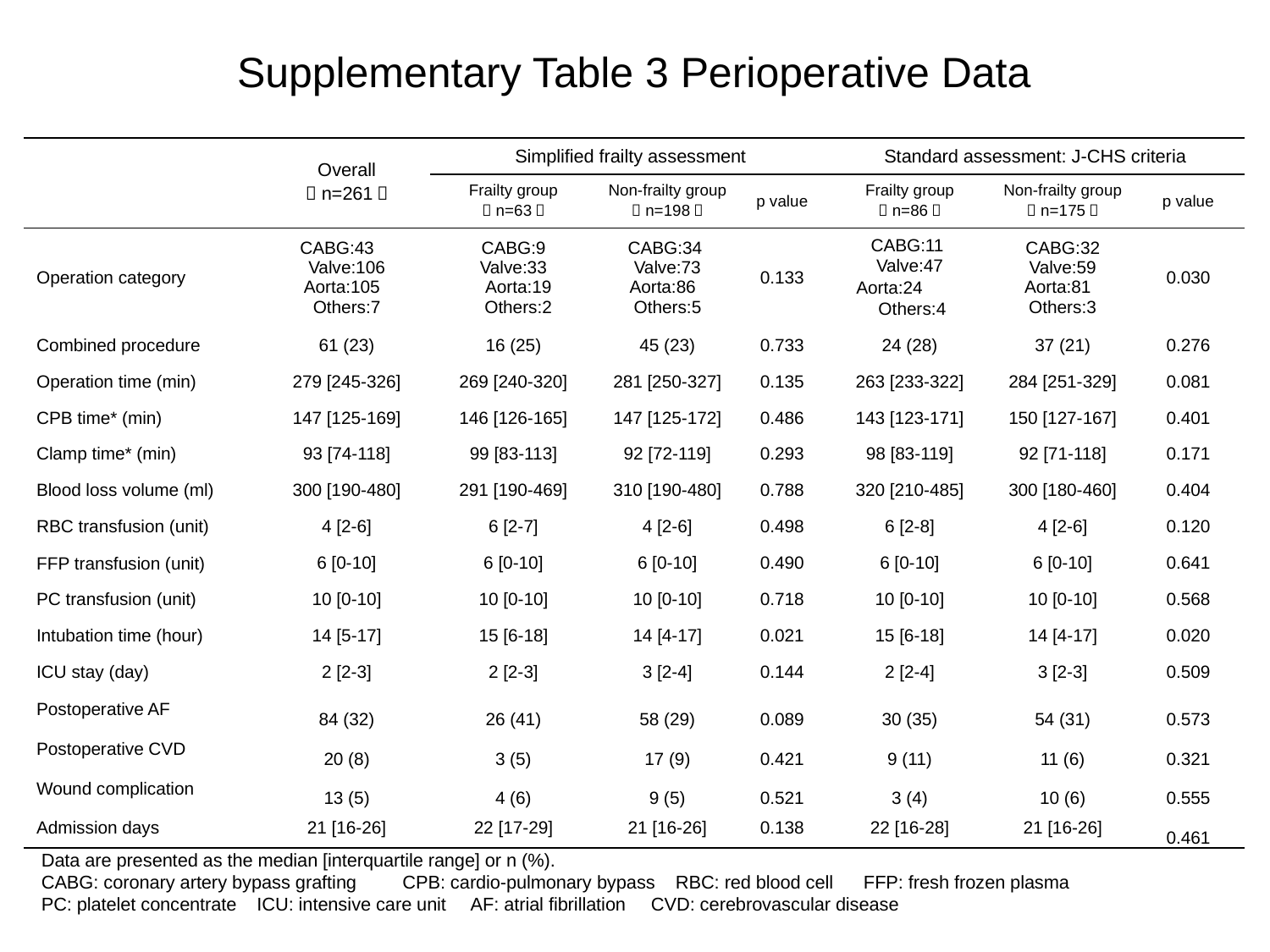

Supplementary Table 3 Perioperative Data
| | Overall （n=261） | Simplified frailty assessment | | | Standard assessment: J-CHS criteria | | |
| --- | --- | --- | --- | --- | --- | --- | --- |
| | | Frailty group （n=63） | Non-frailty group （n=198） | p value | Frailty group （n=86） | Non-frailty group （n=175） | p value |
| Operation category | CABG:43 Valve:106 Aorta:105 Others:7 | CABG:9 Valve:33 Aorta:19 Others:2 | CABG:34 Valve:73 Aorta:86 Others:5 | 0.133 | CABG:11 Valve:47 Aorta:24 　 Others:4 | CABG:32 Valve:59 Aorta:81 Others:3 | 0.030 |
| Combined procedure | 61 (23) | 16 (25) | 45 (23) | 0.733 | 24 (28) | 37 (21) | 0.276 |
| Operation time (min) | 279 [245-326] | 269 [240-320] | 281 [250-327] | 0.135 | 263 [233-322] | 284 [251-329] | 0.081 |
| CPB time\* (min) | 147 [125-169] | 146 [126-165] | 147 [125-172] | 0.486 | 143 [123-171] | 150 [127-167] | 0.401 |
| Clamp time\* (min) | 93 [74-118] | 99 [83-113] | 92 [72-119] | 0.293 | 98 [83-119] | 92 [71-118] | 0.171 |
| Blood loss volume (ml) | 300 [190-480] | 291 [190-469] | 310 [190-480] | 0.788 | 320 [210-485] | 300 [180-460] | 0.404 |
| RBC transfusion (unit) | 4 [2-6] | 6 [2-7] | 4 [2-6] | 0.498 | 6 [2-8] | 4 [2-6] | 0.120 |
| FFP transfusion (unit) | 6 [0-10] | 6 [0-10] | 6 [0-10] | 0.490 | 6 [0-10] | 6 [0-10] | 0.641 |
| PC transfusion (unit) | 10 [0-10] | 10 [0-10] | 10 [0-10] | 0.718 | 10 [0-10] | 10 [0-10] | 0.568 |
| Intubation time (hour) | 14 [5-17] | 15 [6-18] | 14 [4-17] | 0.021 | 15 [6-18] | 14 [4-17] | 0.020 |
| ICU stay (day) | 2 [2-3] | 2 [2-3] | 3 [2-4] | 0.144 | 2 [2-4] | 3 [2-3] | 0.509 |
| Postoperative AF | 84 (32) | 26 (41) | 58 (29) | 0.089 | 30 (35) | 54 (31) | 0.573 |
| Postoperative CVD | 20 (8) | 3 (5) | 17 (9) | 0.421 | 9 (11) | 11 (6) | 0.321 |
| Wound complication | 13 (5) | 4 (6) | 9 (5) | 0.521 | 3 (4) | 10 (6) | 0.555 |
| Admission days | 21 [16-26] | 22 [17-29] | 21 [16-26] | 0.138 | 22 [16-28] | 21 [16-26] | 0.461 |
Data are presented as the median [interquartile range] or n (%).
CABG: coronary artery bypass grafting　　CPB: cardio-pulmonary bypass RBC: red blood cell FFP: fresh frozen plasma
PC: platelet concentrate ICU: intensive care unit AF: atrial fibrillation CVD: cerebrovascular disease

## Slide 5
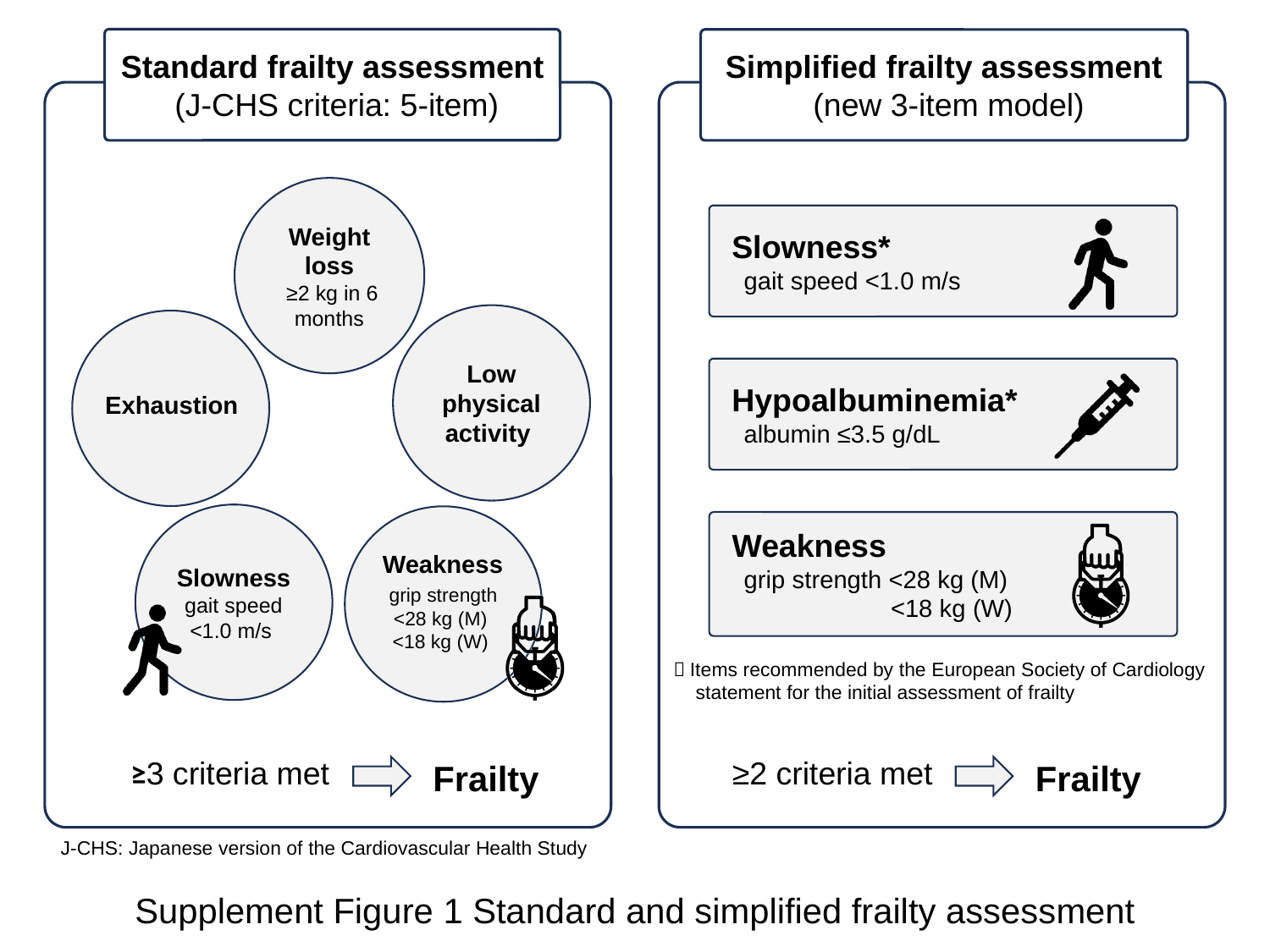

Standard frailty assessment
 (J-CHS criteria: 5-item)
Simplified frailty assessment
 (new 3-item model)
Weight loss
 ≥2 kg in 6 months
 Slowness*
 gait speed <1.0 m/s
Low physical activity
 Hypoalbuminemia*
 albumin ≤3.5 g/dL
Exhaustion
Slowness
gait speed <1.0 m/s
grip strength <28 kg (M) <18 kg (W)
 Weakness
 grip strength <28 kg (M)
 <18 kg (W)
Weakness
＊Items recommended by the European Society of Cardiology
 statement for the initial assessment of frailty
≥3 criteria met
≥2 criteria met
Frailty
Frailty
J-CHS: Japanese version of the Cardiovascular Health Study
Supplement Figure 1 Standard and simplified frailty assessment

## Slide 6
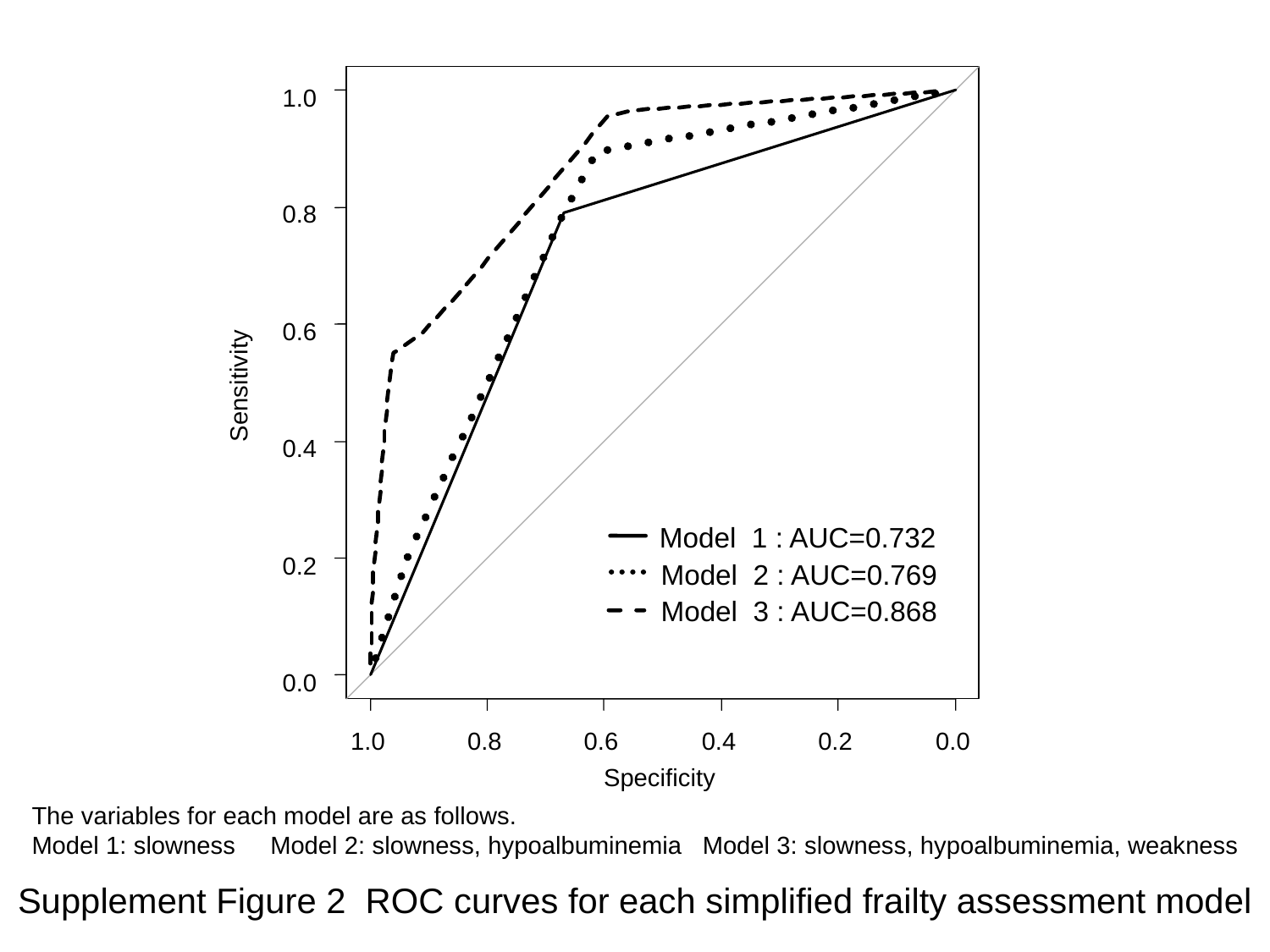

1.0
0.8
0.6
Sensitivity
0.4
Model 1 : AUC=0.732
0.2
Model 2 : AUC=0.769
Model 3 : AUC=0.868
0.0
1.0
0.8
0.6
0.4
0.2
0.0
Specificity
The variables for each model are as follows.
Model 1: slowness Model 2: slowness, hypoalbuminemia Model 3: slowness, hypoalbuminemia, weakness
Supplement Figure 2 ROC curves for each simplified frailty assessment model
